# An Overview of Antibiotics as Emerging Contaminants: Occurrence in Bivalves as Biomonitoring Organisms

**DOI:** 10.3390/ani11113239

**Published:** 2021-11-12

**Authors:** Elena Baralla, Maria P. Demontis, Filomena Dessì, Maria V. Varoni

**Affiliations:** Department of Veterinary Medicine, University of Sassari, 07100 Sassari, Italy; dpiera@uniss.it (M.P.D.); f.dessi@studenti.uniss.it (F.D.); varoni@uniss.it (M.V.V.)

**Keywords:** antibacterial drugs, mollusks, environmental occurrence

## Abstract

**Simple Summary:**

In recent years, the use of antibiotics has increased worldwide in both human and veterinary fields. This led to them accumulating in the environment to such an extent that they are actually included in the category of contaminants of emerging concern. For this reason, many of them have been included in monitoring lists of potential pollutants by competent authorities in order to limit their concentration in surface waters and to determine the risk to the aquatic environments. From this perspective, the aim of this review is to update and discuss the available data on antibiotic residues, using bivalves as biomonitoring organisms. Bivalves are good candidate for this purpose, being globally present in large and accessible populations, sedentary and able to accumulate several xenobiotics thanks to their large filtration capacity. The current research indicates that antibiotics’ presence in bivalves has been investigated along European, American and Asian coasts. Except for tetracycline, determined at high concentration in the North Adriatic Sea, all antibiotics residues in bivalves were under the maximum residual limit established by the competent authorities. Nevertheless, further investigations are necessary in order to prevent antimicrobial resistance, preserve the environment from antibiotic pollution and monitor the associated risk for animals and humans.

**Abstract:**

Antibiotics are used for therapeutic and prophylactic purposes in both human and veterinary medicine and as growth promoting agents in farms and aquaculture. They can accumulate in environmental matrices and in the food chain, causing adverse effects in humans and animals including the development of antibiotic resistance. This review aims to update and discuss the available data on antibiotic residues, using bivalves as biomonitoring organisms. The current research indicates that antibiotics’ presence in bivalves has been investigated along European, American and Asian coasts, with the majority of studies reported for the last. Several classes of antibiotics have been detected, with a higher frequency of detection reported for macrolides, sulfonamides and quinolones. The highest concentration was instead reported for tetracyclines in bivalves collected in the North Adriatic Sea. Only oxytetracycline levels detected in this latter site exceeded the maximum residual limit established by the competent authorities. Moreover, the risk that can be derived from bivalve consumption, calculated considering the highest concentrations of antibiotics residues reported in the analyzed studies, is actually negligible. Nevertheless, further supervisions are needed in order to preserve the environment from antibiotic pollution, prevent the development of antimicrobial resistance and reduce the health risk derived from seafood consumption.

## 1. Introduction

Contaminants of emerging concern (CEC) are chemical substances of recent concern, also detected in several matrices at concentrations higher than expected, with the potential to cause harm to the environment, aquatic life and humans. The largest category of CEC comprises antibiotics and other drugs, such as anti-inflammatories and analgesics [1].

Antibiotics are a group of drugs widely prescribed to treat bacterial infections and with prophylactic purposes in both humans and animals [2]. Furthermore, some of them are used as growth promoting agents in farm animals [3] and aquaculture [4,5]. Antibiotics are considered among the “emerging contaminants” of greatest concern, both because the cumulative toxic effects on aquatic organisms are not well known, and because their continued presence leads to the development of antibiotic resistant bacteria. Moreover, they can act at low concentrations by modifying the natural microbial diversity in aquatic ecosystems [6]. For these reasons, monitoring their presence in the aquatic environment, using appropriate model organisms, is important to assess the risk of these contaminants for the environment and for human health. Given the high use of antibiotics, several monitoring programs have been developed using aquatic biomonitoring organisms. Among them, bivalves have been considered as good bio-indicators of environmental pollution and used in various environmental monitoring programs in coastal waters [7,8,9,10]. Most pharmaceuticals are of polar nature, this can render them directly available to bivalves because of their high bioaccumulation capacities [11].

Bivalves are in fact "filtering" animals, which take in the food suspended in water (microscopic algae, bacteria and detritus) through filter feeding process [12]. They are capable of filtering high volumes of seawater by retaining up to 90% of the particles contained in it, including any contaminant present so as to concentrate them in their tissues directly from water and through diet [13]. Most of them, especially mussels, clams and oysters, have been an important seafood for humans for thousands of years [14] and are still highly popular. They can populate environments of fresh, brackish or salt water, even if only those coming from lagoons and the sea are edible [8]. The majority of them are sedentary species (e.g., mussels and oysters) and spend their lives attached to rocks, wood and other solid objects, whereas others burrow in sand or mud (e.g., clams) or live on the water bottom and swim (e.g., scallops) [6]. Their use in monitoring programs can therefore give information about pollutants’ presence in different areas and marine layers.

Bivalves are among the first animals used by researchers for assessing the environmental quality of seawater [15]. Mussels have been used in biomonitoring programs for systematic measurements on the exposure levels of anthropogenic wastes of many pollutants, from 1975 when Goldberg proposed a world mussel watch [16]. Bivalves have recently been used for the monitoring of pharmaceuticals in coastal waters [9,10]. In addition, they constitute a food source for many other species and can act as transport route of marine pollutants through the food chain [17,18].

One of the advantages of using bivalve mollusks as sentinel organisms is due to their intrinsic characteristics, such as their sedentary life that determines their ability to reflect the quality of the environment around them. Furthermore, they are very resistant organisms, globally present and capable of thriving even in the most unfavorable conditions. Due to their robustness, they can be bred easily and this allows their use for laboratory and field experiments to investigate the effects of different toxic substances and pharmaceutical compounds on aquatic organisms [19,20,21,22]. Moreover, bivalves are also involved in studies analyzing the potential risk of pharmaceutical exposure to humans through the food chain [23]. Furthermore, having a slower metabolism than vertebrates and crustaceans, they are better bio-indicators. Recently, the development and validation of high-performance analytical techniques have made it possible to detect different antibiotic residues at low or very low concentrations in different matrices [24]. Therefore, this review aims to investigate the occurrence of antibiotics in bivalves and the current state of knowledge of antibiotic distribution in the environment all around the world.

We conducted a systematic search for relevant studies using all suitable databases as well as grey literature such as reports and other relevant documents on bivalves’ use as organisms for antibiotic monitoring. In particular, in this review, papers describing the presence of antibiotics in bivalves collected in the last ten years (between 2012 and 2022) all around the world were considered and discussed. We identified 14 studies representing Europe (Italy, Spain and Portugal), Asia (China, Singapore) and America (California, Brazil).

## 2. Antibiotics’ Use and Their Presence in Aquatic Environment

Antibiotics are drugs of natural or synthetic origin and have the ability to kill (bactericidal) or inhibit bacterial growth (bacteriostatic). While the first antibiotics were of natural origin (e.g., penicillin derived from *Penicillium notatum*), currently they are produced in laboratories by chemical synthesis or by semi-synthesis through chemical modification of natural compounds [25]. Antibiotics can be released into the aquatic environment as such or as metabolites from several sources. While 20–30% come from aquaculture, the remaining antibiotics come from the wastewater discharges of industries, hospitals and breeding practices [26,27]. Antibiotics and their metabolites are in fact eliminated through feces and urine and the conventional waste treatment systems are not able to fully eliminate these pharmaceutical compounds [28]. Wastewater treatment plants are important in the lifecycle of antibiotics, such that urban effluent outfalls constitute important sources of these antimicrobial pharmaceuticals into the environment. Surface waters, groundwater and even drinking water can be polluted by antibiotics after irrigation procedures. Moreover, the run off or drainage waters from agriculture and livestock areas are important sources of antimicrobials in the aquatic environment [29]. Once in the environment, antibiotics can be completely degraded or transformed into highly bioactive and potentially toxic substances and they are capable of entering either the aquatic, terrestrial or both, food chains. For this reason, a large amount of antibiotics is found in water with consequent undesirable effects on ecosystems and on human and animal health [30,31,32] including the development of antibiotic resistance [13,33].

Antibiotic resistance is the inability of an antibiotic, administered at therapeutic doses, to reduce survival or inhibit the replication of a pathogenic bacterium [34]. In recent years, this phenomenon has significantly increased due to the overuse and inappropriate use of these drugs. From 2000 to 2015, global antibiotic consumption has increased by 65% and researchers estimate that the total consumption will increase by 202% from 2015 to 2030 without any policy changes [35]. Antibiotic resistance represents a very important public health problem worldwide, as it causes an increase in mortality, prolonged hospital stays and high healthcare costs [36]. It has been shown that antibiotic resistant bacteria can also spread to humans by direct contact with animals and through food [37]. It is estimated that this phenomenon could cause 10 million deaths worldwide by 2050 [38]. The recognition of the risk associated with the presence of antibiotic residues in food of animal origin has led the European Union (EU) to establish maximum residual limits (MRLs) [39,40]. Recently, the EU developed a list of potential water pollutants that should be carefully monitored for up to four years by the EU member states in order to limit the concentration of emerging pollutants in surface waters and to determine the risk they pose to the aquatic environment also considering their potential to bio-accumulate in organisms. In the latest watch list, revisions for four antibiotics were included (amoxicillin, ciprofloxacin, sulfamethoxazole and trimethoprim) [41].

For these reasons, the risk of consuming seafood contaminated with antibiotics even in concentrations below the MRLs should be assessed with caution [38].

**Figure 1:**
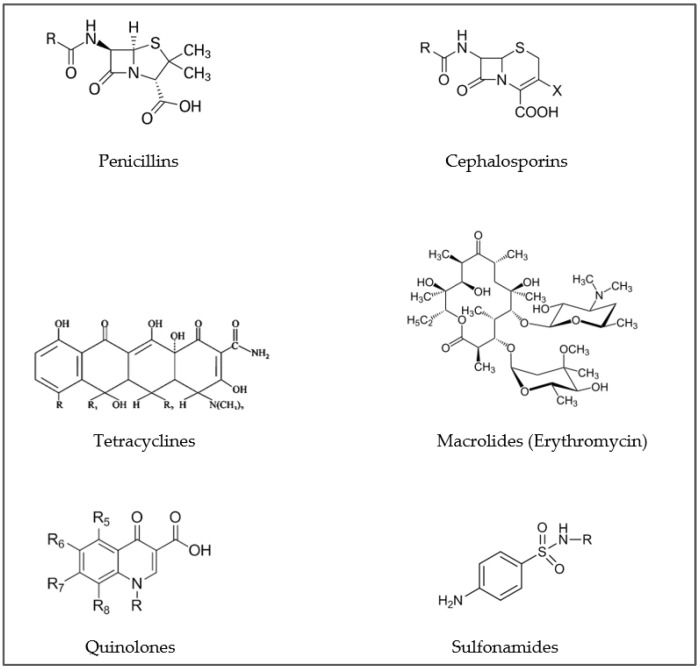
Basic structures of the most used antibiotics in both human and veterinary fields.

According to the World Health Organization (WHO) report on surveillance of antibiotic consumption, the use of the different classes of antibiotics varies in different countries of the world [42]. In four countries in Africa the most used antibiotics in humans are β-lactams, tetracyclines and quinolones, and β-lactams, macrolides and tetracyclines in six countries of America. A high level of consumption of cephalosporins and quinolones were observed in some countries of south east Asian regions and a very high level of consumption of third-generation cephalosporins in all Indian states. β-lactams, macrolides, sulfonamides and tetracyclines were the most used in humans in 45 countries of the EU while β-lactams, macrolides and tetracyclines’ high use was reported in three countries of the Eastern Mediterranean region (Iran, Jordan, Sudan). Finally, β-lactams, tetracyclines and quinolones were the most used in six countries of the Western Pacific region [42]. Data shown in the fifth annual report on antimicrobial agents intended for use in animals, report a prevalence of tetracyclines, penicillins and macrolides in terrestrial food producing animals and amphenicols, tetracyclines and fluoroquinolones in aquatic food producing animals [43]. Figure 1 illustrates the most used antibiotics in both human and veterinary field.

As reported by Zhu et al. (2013), China is the largest producer and user of antibiotics in the world [44]. About half of the antibiotics produced in China end up in animal feed, but their use in aquaculture is limited [45]. From 1996 to 2013 a total of 20 antibiotics belonging to eight different categories (aminoglycosides, β-lactams, chloramphenicols, macrolides, nitrofurans, quinolones, sulfonamides, and tetracyclines) were reported for their use in aquaculture [46]. Some of them (chloramphenicol, ciprofloxacin, erythromycin, and furazolidone) were designed only for humans, while others (amoxicillin, chlortetracycline, gentamycin S, oxytetracycline, penicillin G, streptomycin, sulfamerazine S, and sulfisoxazole) were not authorized in aquaculture or have been banned (i.e., erythromycin) but still used occasionally [47].

## 3. Methods Used to Quantify Antibiotics in Bivalves

The development of new and sensible analytical methods permits quantifying residues of CEC in several matrices. This is the case of antibiotics that are frequently detected in water, sediments and biota [48]. As regards bivalves, they constitute a complex matrix given their high lipids and protein content that can interfere with the analysis [49]. Drugs can bound proteins and peptides, with the consequent need for cleavage of these structures before the analysis [50].

For these reasons, a clean-up step is essential to eliminate possible matrix interferences and obtain an extraction method with good recoveries.

The extraction of antibiotics from bivalves often requires a liquid extraction (sometimes a pressurized liquid extraction, PLE) step followed by a solid phase extraction (SPE) process to concentrate or purify the sample before the analysis [4,6,7,11,27,33,48,51,52,53]. SPE is in fact considered the clean-up stage by excellence for antibiotics residues in mollusks [52].

The extracted samples are generally analyzed using a sensible and sensitive method able to also quantify the analyte if present in small traces with low limits of detection (LOD) and quantification (LOQ). For this purpose, liquid chromatography interfaced with mass spectrometer detection (LC-MS/MS) is actually the most used technique [4,6,11,27,33,48,51,52,53].

As reported in Table 1, the analytical procedures reported in literature that use mass spectrometry detection were able to quantify antibiotics in bivalves with LOD and LOQ values comprised between 0.01 and 1.9 ng/g and 0.02 and 2.11 ng/g, respectively [4,6,7,27,33,48,51,52,53].

## 4. Antibiotics Occurrence in Bivalves Collected All around the World

The concern that pharmaceutically active compounds are able to induce specific biological effects on animals, humans and surrounding environment, lead many scientists to study their occurrence in ecologically relevant seafood species [1,2]. Among pharmaceuticals, antibiotics are frequently detected in several environmental matrices. Table 2 reports the occurrence of antibiotics in bivalves collected all around the world.

### 4.1. Occurrence of Antibiotics in Bivalve Mollusks Harvested in Europe

Several studies report their analyses in bivalves harvested along European coasts [6,7,51,52,54].

In Italy, two studies described the occurrence of antibiotics in bivalves harvested in two different locations of the North Adriatic Sea between 2013 and 2018 [6,51]. Chiesa et al. (2018) analyzed mussels and clams collected from various Food and Agricultural Organization (FAO) marine zones. Both wild and farmed shellfish were collected in this study, in order to evaluate if a possible antibiotic presence could be correlated with treatment in farms or to environmental pollution [6]. Four tetracyclines were detected in farmed clams collected from the Italian side of the North Adriatic Sea (tetracycline, oxytetracycline, doxycycline and chlortetracycline) while no antibiotics were detected in wild shellfish. Moreover, fluoroquinolone flumequine was also found in mussels and clams belonging to farms located in the North Adriatic Sea. Because of the low number of positive samples, no differences could be found between clams and mussels. Moreover, the tetracycline found at the highest concentration by Chiesa et al. (2018) was oxytetracycline, which is also commonly used to fight bacterial diseases because of its wide antibacterial spectrum and its high availability [6]. Álvarez-Muñoz et al. (2015) investigated the presence of several pharmaceutically active compounds in seafood collected in potential contaminated areas of Europe. In Italy, they reported high levels of these compounds in bivalves collected from the Po Delta, where several sources of contamination, such as waste water treatment plants and caring institutes, are located [51]. They investigated the presence of 7 antibiotics and, between them, only azithromycin was found in bivalves at concentrations above the LOD of the used method (found concentration = 13.3 ng/g dw; LOD = 0.01 ng/g dw).

During their study regarding the occurrence of antibiotics in shellfish from various FAO areas, Chiesa et al. (2018) also analyzed mussels collected along the Spanish Atlantic coasts, reporting the presence of tetracycline in a pool of mussels grown in this site. Nevertheless, the analyzed bivalves had been depurated in a plant in North Italy, so that authors speculated that an illegal practice had occurred during the depuration step [6].

In Spain, Álvarez-Muñoz et al. (2015) analyzed bivalves collected between November and December 2013 from the Ebro Delta, an area where important sites for shellfish aquaculture are located [51,59]. Here, mussels (*Mytilus galloprovincialis*), clams (*Chamelea gallina*) and oyster (*Crassostrea gigas*) samples were analyzed: ronidazole levels were detected in clams and oysters (1 ng/g dw and 1.8 ng/g dw, respectively). Furthermore, sulfamethoxazole was found in clams and oysters from the Ebro Delta, but at levels below the LOQ of the method (LOQ = 0.2 ng/g dw). Moreover, the antibiotic azithromycin was found in all shellfish collected in this area with the highest level reported for oysters and mussels [51,52].

As regards the antibiotic ronidazole, it was also detected in bivalve samples collected between October 2016 and March 2017 along the coastline of the Rías Baixas in Spain (Ría de Muros y Noia). This is an important revenue source for the whole of Spain, thanks to the location of several seafood and mussel farms in culture rafts. Moreover, it is a highly populated area served by wastewater treatment plants [7]. In this site, Martínez-Morcillo et al. (2020) investigated the presence of some macrolides and nitroimidazoles determining ronidazole at concentrations of 2.26 ng/g dw in the bivalve *Ensis siliqua*.

Serra-Compte et al. (2021) investigated the presence of sulfonamides, macrolides/β-lactams, tetracyclines and quinolones in mollusk hemolymph sampled in June 2018 from aquaculture structures located in the bays of Alfacs and Fangar on the Mediterranean coast of Spain. Here, two quinolones were detected in mussels’ biofluid: enrofloxacin (0.230 µg/L) and marbofloxacin (0.435 µg/L) [54].

Only one study is actually reported in literature, describing antibiotic occurrence in bivalves harvested in Portugal [51]. Álvarez-Muñoz et al. (2015) analyzed Atlantic mussels collected from one of the largest estuaries in Europe, the Tagus estuary, situated in the most populated area of Portugal near the city of Lisbon. Here two antibiotics were detected: dimetridazole and azithromycin at concentrations of 7.7 ng/g dw and 11.8 ng/g dw, respectively.

### 4.2. Occurrence of Antibiotics in Bivalve Mollusks Harvested in Asia

Asian coasts are, predominantly, the sites were the majority of studies investigating antibiotic occurrence in bivalves have been developed [4,27,33,46,48,53,60,61].

Li et al. (2012) investigated the distribution of quinolones, sulfonamides and macrolides using mollusks sampled in nine coastal sites along the Chinese Bohai Sea [4]. Bohai Bay is a semi-enclosed sea surrounded by important highly populated economic centers and it has been investigated for environmental pollution by several authors [62,63]. In their study, Li et al. (2012) analyzed nine bivalve species collected from late July to early August 2006, 2007 and 2009 for the presence of 22 antibiotics. Between them, antibiotics belonging to the quinolones family were the most frequently and abundantly detected and no significant differences were observed among the three years of sampling [4].

Chen et al. (2015) investigated the presence of sulfonamides, tetracyclines, fluoroquinolones, macrolides and ionophores in biota samples (including fish, shrimps and mollusks) collected in marine aquaculture farms surrounding Hailing Island (South China) in September 2013 [33]. The chosen collection site is one of the most important marine aquaculture zones in China, with wastewaters discharges poured directly into the marine environment. In this study, only erythromycin-H_2_O, salinomycin and its derivative narasin were detected in mollusks at concentrations lower than those reported for the other biota samples analyzed [33].

Zhang et al. (2018) investigated the occurrence of sulfonamides, fluoroquinolones, macrolides and chloramphenicol in the marine environment of the Beibu Gulf located in the South China Sea [27]. It is a semi-closed gulf with characteristics that makes it suitable for breeding several marine organisms, and as such it represents an important mariculture base for the whole of China. As regards bivalves, authors analyzed oyster samples collected in October 2015 in open estuaries. Here, 13 antibiotics were quantified, with the highest concentration found for the macrolide erythromycin (mean concentration = 1.08 ng/g ww) [27].

Another study was developed on the South China coast in order to evaluate the occurrence of antibiotics and other pharmaceuticals and personal care products in the marine environment from the mariculture areas of the Pearl River Delta [53]. It is an economically developed area and highly populated whose activity is strongly based on fishery. Here, several pharmaceuticals were investigated including sulfonamides, quinolones, tetracyclines, β-lactams, aminoglycosides, macrolides, and amphenicols in biota samples including cultured fish and shellfish. *Ostrea gigas*, *Mimachlamys nobilis* and *Mytilus edulis* were collected during autumn 2014 (October) and spring 2015 (March). Of all analyzed antibiotics in biota, norfloxacin resulted in being the most abundant one (31 ng/g ww) followed by spectinomycin (18 ng/g ww). Other antibiotics, such as trimethoprim, tetracycline and cefotaxime, were however also found with a high frequency of detection at low concentrations [53].

Wu et al. (2020) examined quinolone residues in aquaculture bivalves collected from main production districts in Taiwan during June 2018–December 2019. They examined 58 bivalve samples detecting flumequine and enrofloxacin in 3 samples at levels of 0.8 ng/g and 0.5 ng/g, respectively [55].

Zhou et al. (2020) (2021) analyzed blood clams collected in June 2019 from Yueqing Bay, Zhejiang in China. They developed two studies to investigate the effects of microplastics on the bioaccumulation of oxytetracycline and florfenicol (highly used in the veterinary field) in this bivalve species [60] and to deepen the knowledge about the single and combined effects of veterinary antibiotics and microplastics on the immune responses of bivalves [46]. The content of the two tested antibiotics in collected blood clams before the exposure experiment were found to be below the detection limits of 50 µg/kg and 1 µg/kg for oxytetracycline and florfenicol, respectively [46,60].

Similarly, during July 2019, Han et al. (2021) analyzed mussels collected from Shengsi Island in China during a study developed to analyze the immunity response modifications induced by oxytetracycline, florfenicol and sulfamethoxazole alone or in combination with microplastics in mussels. Before the beginning of their experiment, they verified the absence of the three tested antibiotics at concentrations above the detection limit of their method (50 µg/kg, 1 µg/kg and 5 µg/kg for oxytetracycline, florfenicol and sulfamethoxazole, respectively) [61].

Only one study reports the analysis of antibiotics and other emerging contaminants in clams (*Polymesoda expansa*) belonging to the mangrove ecosystem in Singapore, in order to explore the bioavailability of some pharmaceutically active compounds [48]. In local clams, the two antibiotics, sulfadiazine and lincomycin, were found and quantified in 6 sampling sites of mangrove, with the highest concentrations found in the Sungei Mandai site located near an industrial park (1.4 ng/g ww and 0.03 ng/g ww for sulfadiazine and lincomycin, respectively) [48].

### 4.3. Occurrence of Antibiotics in Bivalve Mollusks Harvested in America

To date, four studies are reported in literature investigating the presence of antibiotics in bivalves collected around American coasts in order to evaluate the bioaccumulation of these emerging contaminants in these aquatic organisms and to estimate both environmental contamination and possible adverse effects in shellfish consumers [28,56,57,58].

Klosterhaus et al. (2013) analyzed mussels collected from five sites in San Francisco Bay during December 2009 and January 2010 for their antibiotic content. In detail, they focused on the detection of 32 antibiotics, other personal care product ingredients and alkylphenols. Being highly urbanized, the sampling sites are influenced by several potential contamination sources, such as industrial and municipal wastewater and stormwater runoff [56]. Only two antibiotics among the 32 analyzed, were detected in *Geukensia demissa* samples and, fortunately, at low concentrations as also stated by the authors (0.1 ng/g ww and 0.25 ng/g ww for erythromycin and sulfamethizole, respectively) [56].

Dodder et al. (2014) and Maruya et al. (2014) developed a multiagency pilot study along Californian coasts in order to update the list of contaminants of emerging concern and, eventually, to incorporate them into the Mussel Watch Program teamed by a multi-agency committee [57,58]. In their studies, mussels were collected from 68 sites along the Californian coast between November 2009 and April 2010. Several contaminants of emerging concern were analyzed in this study, including pesticides, alkylphenols, flame retardants, personal care products and pharmaceuticals, such as antibiotics. In particular, five antibiotics were determined in mussels, with the highest mean concentration reached for the fluoroquinolone lomefloxacin (mean 29 ng/g dw) with a detection frequency of 62%. Results obtained by Dodder et al. (2014) and Maruya et al. (2014) suggest that lomefloxacin, and other investigated compounds other than antibiotics, should be included in bivalve monitoring programs, given the high detection frequency and concentrations found [57,58].

Mello et al. (2022) investigated the occurrence of pharmaceuticals, including the antibiotic chloramphenicol that is prohibited for use in foods of animal origin. In this study, two Brazilian coastal areas were chosen as sampling points during July–August 2019: Sepetiba Bay and Parnaiba Delta River [28]. Chloramphenicol was detected at concentrations ranging from values below the LOQ of the method (0.26 ng/g ww) to 0.5 ng/g ww. Despite the low concentrations found, the determination of the prohibited antibiotic arouses the importance of monitoring consumed seafood in Brazil widely [28].

## 5. Discussion and Perspectives

Global antibiotic consumption has been growing because of the increase in human population and the high demand for food containing animal proteins [25]. This led to the introduction of these drugs into the environment so that they are now actually recognized as CEC [24,64,65]. Antibiotics pollution can be derived from both human sources, such as wastewater treatment plant effluents, drug use and hospital wastes, and from veterinary ones. Their administration to food-producing animals and in aquaculture constitutes a high percentage of antibiotic use worldwide [25]. Depending on the physicochemical properties of antibiotics belonging to different classes, they are able to accumulate in several environmental and aquatic matrices posing a serious threat for non-target organisms [24,65]. To evaluate the risk of these emerging contaminants for the environment and human health, it is very important to monitor antibiotic occurrence in the aquatic environment and organisms. To this purpose, bivalves have been known as excellent candidates for biomonitoring programs since 1975 with the first “mussel watch program” [49,66]. Several studies analyzed the bio-accumulative effect of different antibiotics in bivalves using the bio-concentration factor calculated taking into account the concentration of the antimicrobial drugs in biota samples and in water [27,33].

In this review, investigations on the presence of antibiotics in bivalves collected from all around the world have been summarized. Nevertheless, given the high number of antibiotics used in human, veterinary and agricultural fields, not all commercially available antibiotics around the world have been investigated, as can be seen in Table 2. For this reason, it is not easy to formulate a picture of the real global distribution of antibiotics in bivalves. However, this review aims to summarize all data reported in literature for antibiotics residues investigated and detected in bivalves all around the world. According to several studies reported in literature [67,68], a conversion factor from wet weight to dry weight concentration for mussels of 4 to 7 was used to compare all data reported in literature.

**Figure 2:**
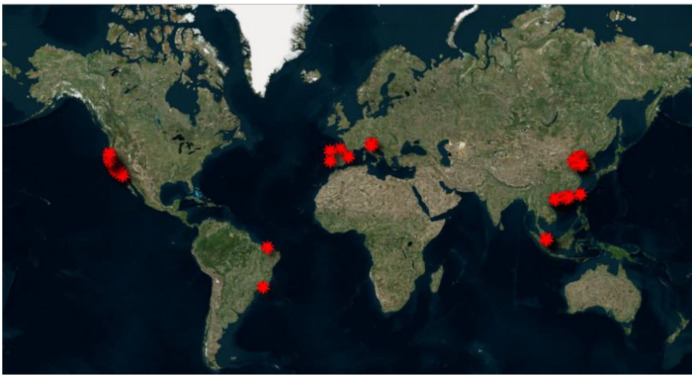
Sampling points of bivalves for antibiotic analyses all around the world.

Figure 2 illustrates the sites where the studies reported in this review have been carried out.

Generally, sampling points were chosen from different authors considering the proximity to contamination sources like hospitals, metropolitan areas, waste water treatment plants, caring institutes and stormwater runoff.

**Figure 3:**
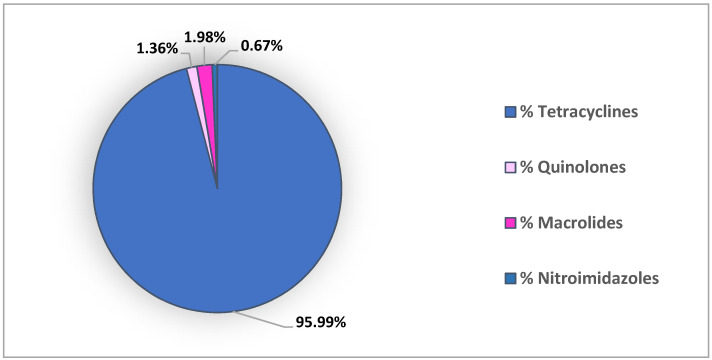
Distribution of antibiotic classes investigated along European coasts.

In Europe, the most frequently detected classes of antibiotics are those of macrolides and nitroimidazoles [7,51] followed by tetracyclines, quinolones [6,54] and sulfonamides [51]. Of all investigated antibiotics in bivalves collected along European coasts (concentrations reported for enrofloxacin and marbofloxacin in mollusks hemolymph were not included in this analysis), the highest residues belong to the tetracycline class, followed by macrolides, quinolones and nitroimidazoles (Figure 3).

The highest concentration was found for oxytetracycline (125.03 ng/g ww) in bivalves belonging to the Italian side of the North Adriatic Sea at concentrations higher than the MRL of 100 µg/kg bw established for fish by the European Commission [69]. This finding is probably correlated with an intentional treatment in farmed bivalves given that oxytetracycline is also commonly used against bacterial diseases at concentrations higher than those recommended in aquaculture [6]. As regards the macrolide azithromycin, it has been detected in Italy, Portugal and Spain at concentrations ranging from about 10 in Italy and Portugal, to 1.3 ng/g dw in Spain. These results are probably related to the Po Delta and Tagus estuary sampling sites, located near contamination sources, such as hospitals and metropolitan areas [51]. Ronidazole deserves a separate discussion. It has in fact been included in the list of prohibited veterinary substances for food-producing animals according to the European Commission [69] and the Codex Alimentarius [70] because of its suspected genotoxic, mutagenic and carcinogenic potential [7]. For this reason, it should not be detected in any food producing animal matrix. Ronidazole concentrations reported by Martinez-Morcillo et al. (2020) and Álvarez-Muñoz et al. (2015) indicate its illegal use in aquaculture or in other food producing activities, such as poultry production. Poultry production is in fact well developed in these areas, being the third (Galicia) and the first (Cataluña) largest poultry producers in Spain [7]. It is therefore conceivable that the illegal use of ronidazole in poultry production would lead to the accumulation of this antibiotic in the aquatic environment including bivalves.

**Figure 4:**
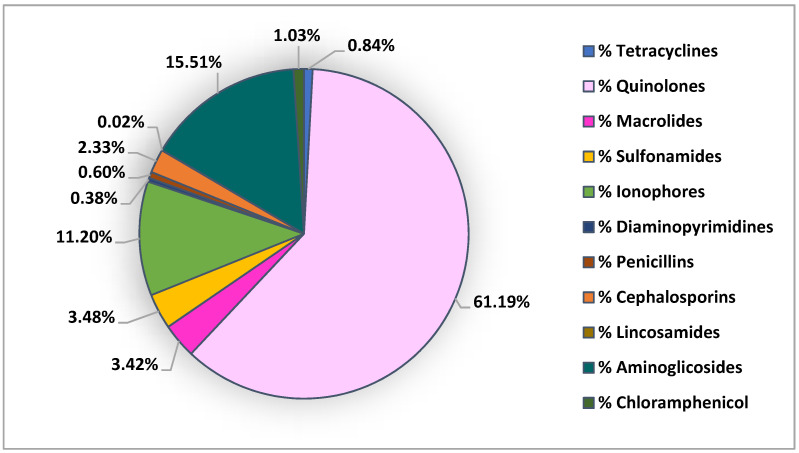
Distribution of antibiotic classes investigated in bivalves collected along Asian coasts.

Several studies reported the occurrence of antibiotic residues in bivalves harvested along Asian coasts [4,27,33,48,53]. On this continent, China is a high producer and consumer of antibiotics with an annual production of about 248,000 tons, 48% of which is used to treat humans, 42% for animals and the remaining 10% is exported [27]. Moreover, according to the United Nations Food and Agriculture Organization, China provides 90% of the total aquaculture products in the world [25,71]. For this reason, it is of great importance to monitor antibiotic presence in the aquatic environment given that they are commonly used to combat diseases and achieve higher economic benefits in aquaculture [53]. In mussels found along Asian coasts, several classes of antibiotics were revealed: quinolones, sulfonamides, macrolides, ionophores, diaminopyrimidines, tetracyclines, penicillins, cephalosporins, aminocyclitols, lincosamides and chloramphenicol (Figure 4).

The highest concentration between all investigated antibiotics in bivalves collected along Asian coasts was reported by Xie et al. (2019) for the quinolone norfloxacin in biota composed from both fish and shellfish samples, belonging to the Pearl River Delta. In this study, the levels of these emerging contaminants were significantly higher in *Mytilus edulis* and *Ostrea gigas* than in fish and carnivorous shellfish [53]. Furthermore chloramphenicol was detected by Xie et al. (2019) in *Mytilus edulis* collected from Zhuhai Guishan Island in 2014 and authors blamed this on environmental pollution, considering that chloramphenicol has been banned for its use in animal breeding in China since 2002 [53,72]. In general, sulfonamides, quinolones and macrolides were the most frequently detected classes of antibiotics along Asian coasts.

**Figure 5:**
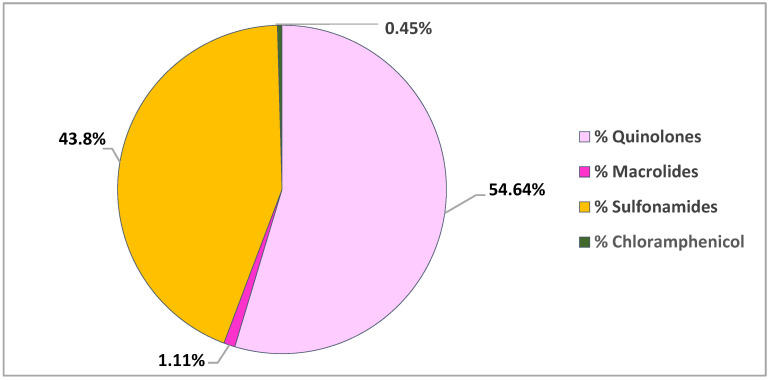
Distribution of antibiotic classes investigated in bivalves collected along American coasts.

The four studies reported for antibiotic residues in bivalves collected in America detected the presence of quinolones, macrolides and sulfonamides along Californian coasts and chloramphenicol in Brazil [28,56,57,58] (Figure 5).

Between all investigated antibiotics in bivalves collected along American coasts, the highest mean concentrations were reported for the quinolone lomefloxacin and the sulfonamide sulfamethazine that were found in similar concentrations (29 ng/g dw and 24 ng/g dw for lomefloxacin and sulfamethazine, respectively) in *Mytilus* spp. Moreover, the occurrence of these antibiotics was particularly evident in sites where agricultural and veterinary activities were highly developed [58].

The global distribution of antibiotics in bivalves collected all around the world revealed that the most frequently detected classes of antibiotics in bivalves were macrolides followed by sulfonamides and quinolones (Table 2).

The highest concentration between all investigated antibiotics was reported for tetracyclines residues detected in bivalves collected in the North Adriatic Sea. Nevertheless, this cannot exclude a contamination from non-investigated antibiotics in bivalves.

Given the high consumption of seafood all around the world, it is essential to evaluate the associated human health risk. For this purpose, the estimated daily intake (EDI; μg/kg bw/d) should be calculated taking into account the average consumption of seafood among people belonging to different countries. The EDI can be calculated according to the following formula:

EDI = C × DC/BW(1)

where C (ng/g ww) is the residual concentration of antibiotics determined in bivalves, DC is the mean daily consumption of seafood (g/d) and BW is the average body weight of consumer (kg bw).

Considering the mean concentration of tetracyclines detected in Europe (62.6 ng/g ww) that represent the highest concentration of investigated antibiotics revealed in bivalves all around the world, the mean consumption of mollusks in occidental countries [73] and the mean body weight for an adult (70 kg for an adult in occidental countries) the EDI value is 5.4 × 10^−3^ μg/kg bw/d. The same parameter calculated in Asian countries, where a high consumption of mollusks is recorded [27,74], for the quinolone norfloxacin, which was the one detected at the highest concentration of all investigated antibiotics in Asian bivalves (31 ng/g ww) and considering a mean body weight for an adult of 60 kg, is 1.7 × 10^−2^ µg/kg bw/d.

In these cases, the hazard quotient expressed as the ratio between the EDI and the admissible daily intake (ADI) calculated as reported by Martínez-Morcillo et al. (2020), is 0.0002 and 0.001 for tetracycline and norfloxacin, respectively. These values indicate a negligible risk of <0.1 [7].

Nevertheless, the risk that can derive from antibiotic residues in bivalve consumed by humans is influenced by several factors, such as physiological/pathological conditions, age and dietary habits. Moreover, antibiotic residues can also be assumed to be from other food sources so that the daily intake of these emerging contaminants can be higher than that calculated.

## 6. Conclusions

To conclude, except for oxytetracycline in bivalves belonging to the North Adriatic Sea, all the studies reported in this review revealed antibiotics residues under the MRLs defined by the competent authorities. For this reason, constant monitoring programs of antibiotic residues in bivalves should be developed. Moreover, other antibiotics should also be investigated in bivalves collected all round the world, in order to have a more exact picture of their distribution as emerging contaminants.

Further supervisions are therefore needed to preserve the environment from antibiotics pollution, prevent the high concern of the development of antimicrobial resistance and to evaluate and monitor the health risks derived from seafood consumption.

## Figures and Tables

**Table 1:** Methods used to quantify antibiotics in bivalve mollusks.

Antibiotic Class	Extraction Method	Detection	Recovery %	LOD (ng/g dw)	LOQ (ng/g dw)	Reference
Quinolones Sulfonamides Macrolides	PLE+SPE	LC-MS/MS	n.d.	0.1–0.3 0.02–0.05 0.04–0.6	0.3–0.9 0.06–0.6 0.1–1	[4]
Tetracyclines Quinolones	LE+SPE	LC-MS/MS	92–113 89–91	0.51–1.26 * 0.54 *	0.65–1.48 * 0.83 *	[6]
Sulfonamides Quinolones Macrolides	SPE	UPLC-MS-MS	67–85 70–93 63–80	0.02–0.03 * 0.06–0.16 * 0.01–0.25 *	0.06–0.10 * 0.21–0.54 * 0.02–0.85 *	[27]
Macrolides Ionophores	LE-SPE	RRLC-MS/MS	76.5–379.2 53.42–366.5	0.23–0.63 *	0.76–2.11 *	[33]
Nitroimidazole	PLE+SPE	UPLC–QqLIT	n.d.	0.2	0.7	[7]
Sulfonamides Lincosamides	LE	LC-MS/MS	61–131 51	0.01–0.03 0.01	n.d.	[48]
Macrolides Nitroimidazoles Sulfonamides	PLE+SPE	UPLC-MS/MS	45–54.4 65.8–66 34.7–41.3	0.01 0.01–0.10 0.01–0.03	0.02–0.03 0.03–0.33 0.02–0.09	[51,52]
Sulfonamides Diaminopyrimidines Quinolones Tetracyclines Penicillins Cephalosporins Macrolides Chloramphenicol	LE+SPE	UHPLC-MS/MS	43–127	0.01–0.05 * 0.01 * 0.01–1.9 * 0.03–0.3 * 0.01 * 0.09 * 0.03–0.3 * 0.2 *	0.03–0.2 * 0.03 * 0.03–5.6 * 0.1–0.9 * 0.03 * 0.3 * 0.1–1 * 0.6 *	[53]

n.d. = not determined; * ng/g ww; LC-MS/MS = liquid chromatography–tandem mass spectrometry; UHPLC-MS/MS = ultra-high-pressure liquid chromatography tandem mass spectrometry; RRLC-MS/MS = rapid resolution liquid chromatography/tandem mass spectrometry; UHPLC-QqLIT = ultra-high-pressure liquid chromatography quadrupole-linear ion trap mass spectrometry.

**Table 2:** Concentrations of antibiotics (ng/g) in bivalve mollusks harvested in the World.

Continent	State-Country	Antibiotic	Location	Mollusk	Analytical Method Used	Antibiotic Concentration Found (ng/g dw)		Reference
						Mean	Range	
Europe	Italy	Tetracycline	North Adriatic Sea	*Meretrix lyrata*	LC-MS/MS	49.45 *	n.d.	[6]
		Oxytetracycline		*Venerupis*		125.03 *	n.d.	
		Doxycycline		*decussata*		60.45 *	n.d.	
		Chlortetracycline		*Venerupis philippinarum*		77.48 *	n.d	
		Flumequine				0.84 *	n.d.	
				*Meretrix meretrix*				
				*Paphia textile*				
				*Venus gallina*				
		Flumequine		*Mytilus*		3.59 *	n.d.	
				*galloprovincialis*				
				*Mytilus edulis*				
				*Mytilus chilensis*				
		Azithromycin	Po Delta	*Mytilus galloprovincialis*	UPLC-MS/MS	13.3	13–13.6	[51]
	Spain	Tetracycline	Atlantic Spain	*Mytilus* *galloprovincialis* *Mytilus edulis* *Mytilus chilensis*	LC-MS/MS	0.55 *	n.d.	[6]
		Ronidazole	Ebro Delta	*Chamelea gallina*	UPLC-MS/MS	1	n.d.	[51]
				*Crassostrea gigas*		1.8	n.d.	
		Sulfamethoxazole		*Chamelea gallina*		<0.02	n.d.	
				*Crassostrea gigas*		<0.02	n.d.	
		Azithromycin		*Mytilus galloprovincialis*		2.9	n.d.	
				*galloprovincialis*				
				*Chamelea gallina*		1.3	n.d.	
				*Crassostrea gigas*		3	n.d	
		Ronidazole	Ría de Muros y Noia	*Ensis siliqua*	UPLC–QqLIT	2.26	n.d.	[7]
		Enrofloxacin	Alfacs Bay Mediterranean Sea	*Mytilus galloprovincialis*	UHPLC-QqLIT	0.230 ^▲^	0.198–0.208 ^▲^	[54]
		Marbofloxacin				0.435 ^▲^	0.424–0.446 ^▲^	
	Portugal	Dimetridazole	Tagus estuary	*Mytilus* spp.	UPLC-MS/MS	7.7	10.2–5.2	[51]
		Azithromycin				11.8	12–11.6	
Asia	China	Norfloxacin	Coastal cities along the Bohai Sea: Dalian, Yingkou, Huludao, Beidaihe, Tianjin, Shouguang, Penglai, Yantai and Weihai	*Crassosteratalienwhanensis* *Chlamys farreri*, *Amussium*, *Scapharca subcrenata*, *Meretrix merehjgntrix* *Linnaeus,* *Mactra veneriformis*, *Mactra chinesis*, *Mya arenaria*, *Neverita didyma*, *Rapana venosa,* *Mytilus edulis*	LC-MS/MS	18.82	0–370	[4]
		Ciprofloxacin				14.54	0–208	
		Difloxacin				3.14	0–57.1	
		Enrofloxacin				5.43	0–147	
		Fleroxacin				13.57	0–250	
		Ofloxacin				14.65	0–242	
		Lomefloxacin				5.67	0–141	
		Sarafloxacin				10.94	0–160	
		Sulfathiazole				1.33	0–35.2	
		Sulfamethoxazole				1.01	0–20.1	
		Sulfisoxazole				0.91	0–71.6	
		Sulfadiazine				0.16	0–2.72	
		Sulfapyridine				0.26	0–3.65	
		Sulfadimethoxine				0.08	0–1.75	
		Sulfamethazine				0.2	0–29.8	
		Sulfamerazine				0.42	0–5.98	
		Sulfamonomethoxine				1.60	0–15.4	
		Spiramycin				0.78	0–23.2	
		Josamycin				0.03	0–1.14	
		Tylosin				0.01	0–1.81	
		Erythromycin				1.65	0–31.3	
		Roxithromycin				0.11	0–1.92	
		Erythromycin-H_2_O	Hailing Island of South China	*Meretrix lusoria*	RRLC-MS/MS	n.d.	n.d.–0.9 *	[33]
		Salinomycin				13 *	n.d.–14.5 *	
		Narasin				n.d	n.d.–7.5 *	
		Sulfadiazine	Dalang, Jingu, Dafeng and Nanliu River estuaries–Beibu Gulf	*Crassostrea rivularis Gould*	UPLC-ESI-MS/MS	0.12 *	n.d.	[27]
		Sulfapyridine				0.45 *	n.d.	
		Sulfamethazine				0.03 *	n.d	
		Sulfamethoxazole				0.03 *	n.d.	
		Trimethoprim				0.14 *.	n.d.	
		Norfloxacin				0.37 *	n.d.	
		Ciprofloxacin				0.54 *.	n.d.	
		Enrofloxacin				0.17 *	n.d.	
		Ofloxacin				0.14 *	n.d.	
		Enoxacin				0.27 *	n.d.	
		Clarithromycin				0.06 *	n.d.	
		Roxithromycin				0.11 *	n.d.	
		Erythromycin				1.08 *	n.d.	
		Sulfamerazine	Pearl River Delta	*Ostrea gigas* *Mimachlamys nobilis* *Mytilus edulis*	UHPLC-MS/MS	0.03 *	n.d.–0.9 *	[53] ^#^
		Sulfamethazine				0.07 *	n.d.–2.1 *	
		Sulfamethoxazole				1.6 *	n.d.–26 *	
		Trimethoprim				0.3 *	0.04–1 *	
		Ciprofloxacin				9.1 *	n.d.–42 *	
		Norfloxacin				31 *	n.d.–256 *	
		Ofloxacin				3 *	n.d.–72 *	
		Flumequine				8.8 *	n.d.–118 *	
		Tetracycline				0.5 *	0.03–2.4 *	
		Oxytetracycline				0.4 *	n.d.–1.8 *	
		Isochlortetracycline				0.08 *	n.d.–0.6 *	
		PenicillinG-Na				0.7 *	n.d.–3.4 *	
		Cefotaxime-Na				2.7 *	0.4–13 *	
		Spectinomycin				18 *	n.d.–366 *	
		Roxithromycin				0.1 *	n.d.–1.8 *	
		Erythromycin-H_2_O				0.9 *	n.d.–5.5 *	
		Clarithromycin				1.2 *	n.d.–23 *	
		Chloramphenicol				1.3 *	n.d.–37 *	
		Enrofloxacin Flumequine	Taiwan: Changhua, Yunlin, Chiayi, Tainan, Kaohsiumg, Hwalien	*Corbicula fluminea* *Meretrix lusoria*	LC-MS/MS	0.5 * 0.8 *	n.d. n.d.	[55]
	Singapore	Sulfadiazine Lincomycin	Mangrove sites: Sungei Mandai Sungei Buloh Wetland Reserve Pandan Mangrove Sungei Changi Pulau Ubin Pasir Ris	*Polymesoda expansa*	LC-MS/MS	0.51 * 0.02 *	0.15–1.4 * 0.01–0.03 *	[48]
America	California	Erythromycin-H_2_O Sulfamethizole	San Francisco Bay:	*Geukensia demissa*	LC-MS/MS	0.1 * <0.25 *	<0.06–0.1 * <0.25–0.2 *	[56]
			Richmond					
			San Leandro Bay					
			Eden Landing					
			Foster City					
			Cooley Landing					
		Lomefloxacin	Coast of California	*Mytilus* spp.	LC-MS/MS	29	0–170	[57,58]
		Sulfamethazine				24	0–430	
		Enrofloxacin				1.3	0–12	
		Erytromycin-H2O				0.14	0–2	
		Ofloxacin				1.2	0–18	
	Brazil	Chloramphenicol	Sepetiba Bay and Parnaiba Delta River	*Anomalocardia*	LC-MS/MS	<0.26 *	n.d.–0.5 *	[28]
				*Brasiliana*				
				*Mytilus edulis*				

n.d. = not determined; * ng/g ww; ^▲^ µg/L hemolymph; ^#^ this study analyzed biota samples that comprised cultured fish and shellfish.

## Data Availability

Not applicable.
